# Impact of Immunosuppression on Immune Cell Dynamics in COVID-19: A Serial Comparison of Leukocyte Data in Healthy and Immunocompromised Patients Before and After Infection

**DOI:** 10.3390/jcm14093223

**Published:** 2025-05-06

**Authors:** Masumi Ogawa, Yasufumi Suzuki, Yusuke Nishida, Daisuke Ono, Hiromi Kataoka, Kyosuke Takeshita

**Affiliations:** 1 Department of Clinical Laboratory, Saitama Medical Center, Saitama Medical University, Kawagoe 350-8550, Saitama, Japan; masumi.o0806@gmail.com; 2 Department of Internal Medicine, Self-Defense Forces Central Hospital, Setagaya-ward 154-8532, Tokyo, Japan; 3 Medical Informatics Room, Saitama Medical Center, Saitama Medical University, Kawagoe 350-8550, Saitama, Japan; 4 Department of General Internal Medicine, Saitama Medical Center, Saitama Medical University, Kawagoe 350-8550, Saitama, Japan; 5 Department of Infectious Diseases and Infection Control, Saitama Medical Center, Saitama Medical University, Kawagoe 350-8550, Saitama, Japan; 6 Faculty of Health Science and Technology, Kawasaki University of Medical Welfare, Kurashiki 701-0193, Okayama, Japan

**Keywords:** COVID-19, immunosuppression, cell population data, white blood cell differential fluorescence scattergram

## Abstract

**Background**: The significance of cell population data (CPD) and leukocyte scattergrams in COVID-19 has not been fully established, partly due to the absence of serial leukocyte monitoring before and after SARS-CoV-2 infection. This study first examined changes in these parameters in non-immunosuppressed subjects over the course of infection. Subsequently, these findings were compared with those observed in patients who were immunosuppressed to assess the impact of immunosuppression. **Methods**: In total, 48 patients with COVID-19 were analyzed. Complete blood count (CBC) results and CPD were assessed using a Sysmex XN-9000 hematological analyzer. **Results**: The control and IST groups had similar clinical characteristics regarding COVID-19 severity and baseline CBC and CPD. WBC and neutrophil counts showed no significant changes immediately post onset; however, they decreased in the control group and increased in the IST group. Platelet counts decreased transiently on days 3–5 in both groups. The control group’s lymphocyte counts significantly dropped, but their lymphocyte-related CPD remained unchanged. The IST group experienced delayed lymphocyte recovery and showed reduced DNA/RNA content and cell size diversity. Scattergrams immediately after onset showed an increase in lymphocyte clusters, particularly juvenile lymphocytes, in the control group, while they decreased in the IST group. In the control group, mature neutrophils decreased while immature neutrophils increased. Conversely, the percentage of mature neutrophils increased in the IST group. Both groups showed minimal plasmacytoid lymphocyte clusters after onset. **Conclusions**: Immunosuppression impairs juvenile cell mobilization, which may increase susceptibility to viral impacts and potentially worsen prognosis by increasing the risk of infection.

## 1. Introduction

Individuals with compromised immune systems are at high risk of contracting severe acute respiratory syndrome coronavirus 2 (SARS-CoV-2) infection and experiencing severe outcomes, including hospitalization and mortality [1]. This group encompasses patients with cancer and those receiving immunosuppressive treatments for autoimmune diseases. Despite vaccination efforts, these individuals were disproportionately affected during the COVID-19 pandemic [2]. Worse hospital outcomes have been observed among adults with cancer and solid organ transplants, potentially due to a higher burden of comorbidities [3]. Investigating the differences in immune responses to SARS-CoV-2 infection between patients not undergoing immunosuppressive therapy and those who are may provide insights into the mechanisms underlying their poor prognoses.

A Sysmex XN-9000 automated hematological analyzer (Sysmex, Kobe, Japan) used flow cytometry to generate white blood cell (WBC) differential fluorescence (WDF) scattergrams and cell population data (CPD). CPD parameters provide quantitative insights into the morphological and functional characteristics of leukocytes, including internal complexity, nucleic acid content, and cell size. Additionally, CPD offers dispersion metrics that reflect the heterogeneity within cell populations. These parameters have been reported to reflect immune cell activation, maturation, and diversity, which are particularly relevant in the context of COVID-19 [4]. By analyzing CPD, this study aimed to detect qualitative differences in immune responses, especially in patients who are immunocompromised, where leukocyte recruitment and functionality may be altered. Abnormal lymphocyte clusters in the WDF scattergrams and changes in CPD can diagnose SARS-CoV-2 infection [5,6]. These tools can help elucidate immune response alterations in patients with immunosuppression. However, previous leukocyte flow cytometry studies have primarily compared patients with COVID-19 with patients who are immunosuppressed in a cross-sectional manner rather than through serial monitoring. This lack of longitudinal data has made it difficult to interpret immune response dynamics at the individual level. To our knowledge, no previous study has evaluated serial changes in leukocyte CPD and WDF scattergrams before and after COVID-19 onset in the same individuals. Moreover, the present study is the first to compare these longitudinal changes between patients who received immunosuppressive therapy for underlying diseases and those who did not.

This study aimed to elucidate the mechanisms underlying reduced immune response and poor prognosis in patients who are immunocompromised. In this analysis, serial changes in CPD and WDF scattergrams before and after COVID-19 onset were compared between patients who did not receive immunosuppressive therapy for their underlying diseases (control group) and those who did (IST group).

## 2. Materials and Methods

### 2.1. Patients

**Figure 1:**
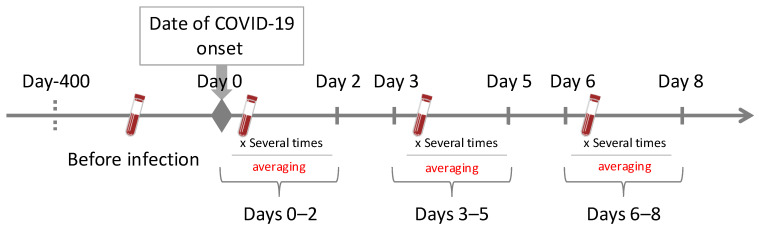
Complete blood count result and cell population data extract procedure. The data collection lasted from 400 days before COVID-19 onset (day-400) to 8 days post onset. The timeline emphasizes key intervals: Day 0 represents the COVID-19 onset date, marking the start of the post-onset analysis period. “Before” refers to the most recent single blood sample collection performed within 400 days before day 0. Post-onset data are grouped into the following periods: days 0–2, days 3–5, and days 6–8. The average values of all blood samples collected during the respective periods are used for these intervals.

All patients diagnosed with COVID-19 via reverse transcription polymerase chain reaction (RT-PCR) at the Saitama Medical Center between 6 April 2020 and 22 October 2022 who were admitted for treatment and who underwent blood sample collection within 7 days of symptom onset were identified from the medical records. The onset date was defined as the first day of upper respiratory symptoms such as fever or cough. In asymptomatic or unclear cases, the date of diagnosis by RT-PCR was used as the onset date. Figure 1 shows the data collection method. Briefly, patients who underwent peripheral blood tests within 400 days before admission were included.

They were categorized into a control group (no immunosuppressive treatment) and an IST group (patients with solid tumors, hematologic malignancies, inflammatory or autoimmune diseases, or organ transplants receiving immunosuppressants, immunomodulators, or steroids). The background diseases and immunosuppressive therapies in the IST group are summarized in Table 1.

The exclusion criteria were as follows: (1) patients who received blood transfusions or granulocyte-colony stimulating factor preparations during the clinical course during COVID-19 onset; (2) patients with concurrent bacterial infections during COVID-19 onset; and (3) patients with pre-existing abnormal white blood cell counts caused by underlying illnesses.

Upon admission, the following parameters were compared: age, sex, disease severity (with or without an oxygen requirement), and comorbidities (hypertension, diabetes, obesity [body mass index ≥ 25 kg/m^2^], chronic obstructive pulmonary disease, asthma, cardiovascular disease, and chronic kidney disease). Disease severity was categorized as mild or severe. Mild cases were defined as the absence of respiratory symptoms or pneumonia findings, with oxygen saturation > 93% in room air and no requirement for oxygen therapy. Severe cases were defined as respiratory failure, an oxygen saturation level < 93% in room air, and a clinical requirement for oxygen administration [7]. Several severity-related variables based on blood test results obtained at the time of admission, including markers of systemic inflammation (C-reactive protein [CRP], procalcitonin, and ferritin), lactate dehydrogenase, aspartate aminotransferase, alanine aminotransferase, and the estimated glomerular filtration rate (eGFR), were also assessed (Table 2 and Table 3). These inflammatory biomarkers were quantitatively measured at the time of admission using standard clinical laboratory analyzers routinely employed in the hospital. The analysis used the single values measured at admission. For serum preparation, blood collection tubes were left to stand for 15 min and then centrifuged at 2270× *g*. Plasma samples were obtained using tubes containing 3.2% sodium citrate (mixed at a 1:9 ratio with blood), followed by centrifugation at 2000× *g* for 10 min. For complete blood count (CBC) and fractionation, samples were collected in tubes containing ethylenediaminetetraacetic acid (EDTA). Inflammatory markers, including CRP, and other biochemical parameters were measured using a Labospect 008 α^®^ analyzer (Hitachi High-Tech Co., Tokyo, Japan). Procalcitonin and ferritin were measured using the Roche COBAS 8000^®^ system (Roche Diagnostics, Basel, Switzerland). CBC and cell population data (CPD) were analyzed using a Sysmex XN-9000^®^ hematology analyzer (Sysmex Co., Kobe, Japan). All parameters were derived from single-time-point measurements and were not serially tracked.

### 2.2. CPD and WBC Differential Fluorescence (WDF) Scattergram

Complete blood count (CBC) parameters, CPD, and WBC differential fluorescence (WDF) scattergrams were analyzed using a Sysmex XN-9000^®^ automated hematology analyzer (Sysmex Corporation, Kobe, Japan). White blood cells were classified into various types of leukocytes, with signals plotted on a three-axis WDF scattergram [8]. The signals obtained from the three axes after preincubation with unique surfactant reagents and fluorescence staining were analyzed and calculated according to the distribution width. Leukocyte differentiation was based on cellular granularity (side-scattered light, the x-axis), nucleic acid/protein content (fluorescent light intensity, the y-axis), and cell size (forward-scattered light, the z-axis). Table 4 depicts the morphological and functional characteristics of the whole CPD panel measured using Sysmex XN-9000. Briefly, the cellular characteristics of neutrophils (NE), lymphocytes (LY), and monocytes (MO) were reported according to their complexity (e.g., NE-WX), cellular content (e.g., NE-SFL), and size (e.g., NE-FSC) in arbitrary units of light scattering [9].

**Figure 2:**
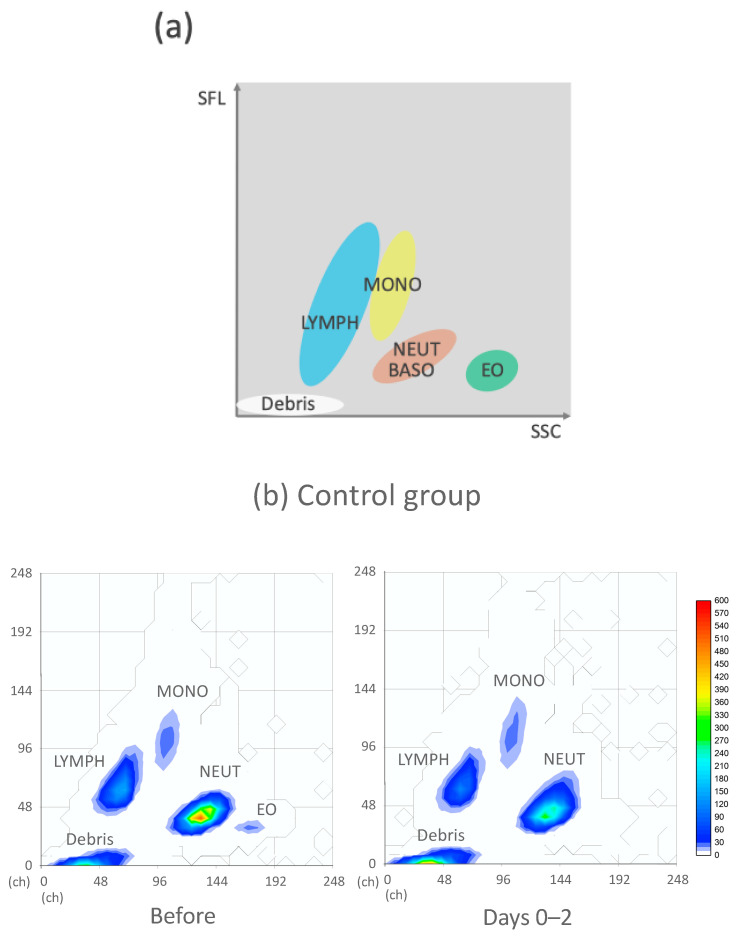

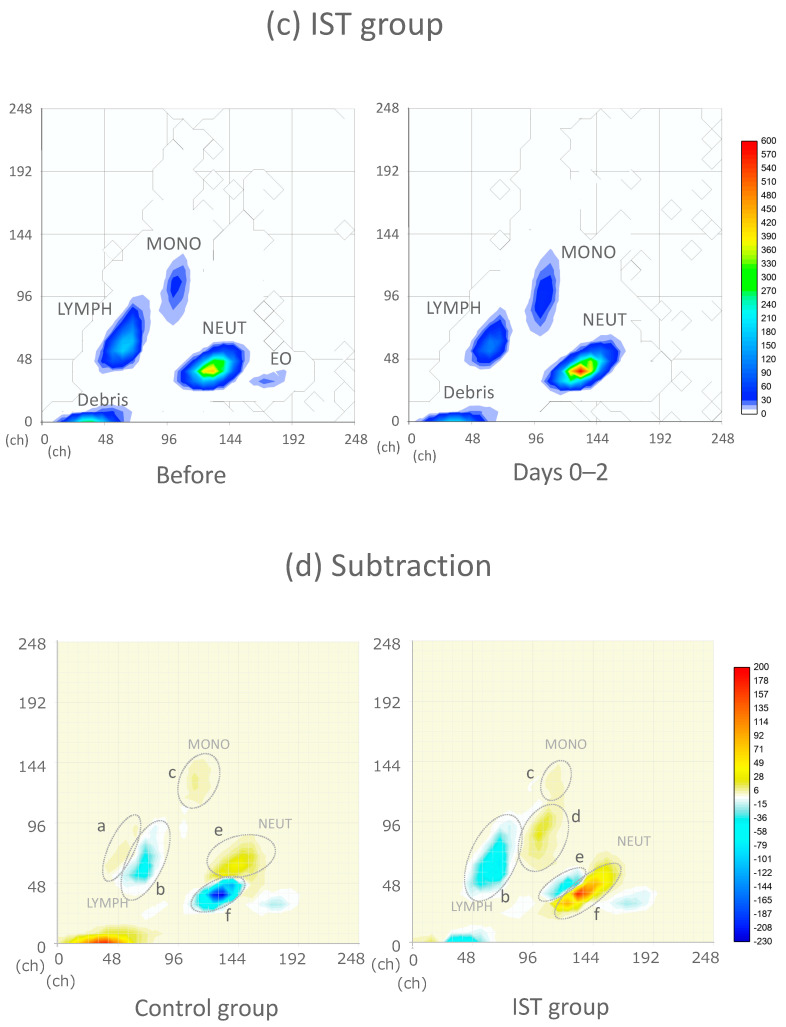
The WDF scattergrams between the control and IST groups before and immediately after COVID-19 infection. In the WDF scattergram, the x-axis (SSC) represents side-scattered light intensity, which reflects cell granularity; the y-axis (SFL) indicates fluorescence intensity, corresponding to nucleic acid content; and the z-axis represents the frequency distribution of cell counts. (**a**) Scattergram legend: blue = lymphocytes, yellow = monocytes, red = neutrophils and basophils, green = eosinophils. (**b**) Comparison of scattergrams before and immediately after COVID-19 onset in the control group (left: before onset, right: days 0–2). (**c**) Comparison of scattergrams before and immediately after COVID-19 onset in the IST group (left: before onset, right: days 0–2). (**d**) Subtraction images showing changes before and after onset in both the control and IST groups. Regions: a = immature lymphocytes, b = mature lymphocytes, c = immature monocytes, d = mature monocytes, e = immature neutrophils, f = mature neutrophil clusters.

**Figure 3:**
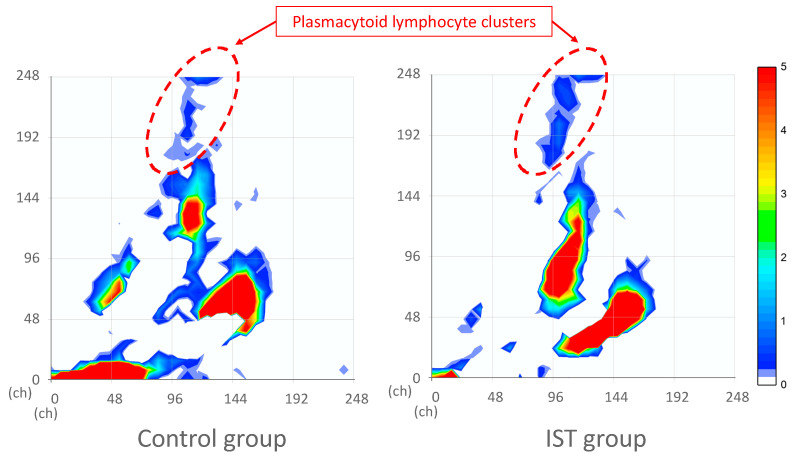
Subtraction images with adjusted scaling to detect plasmacytoid lymphocyte clusters. This figure is based on the subtraction images shown in Figure 2d, with the color scale adjusted to enhance the visibility of plasmacytoid lymphocytes. The scaling was modified to enable the visualization of smaller cell populations. Subtraction images comparing pre-onset and post-onset (days 0–2) data are shown for the control group (**left**) and IST group (**right**). Red dashed lines indicate clusters of plasmacytoid lymphocytes.

The WDF scattergram results of each group were averaged before and after onset, and the following image processing was performed, as shown in Figure 2 and Figure 3. In the WDF scattergram, each 8 × 8 dot segment was treated as a unit block, and these blocks were mapped onto a 32 × 32 matrix. The intensity within each block was aggregated, and the mean intensity of each block across different case groups was calculated. The distribution of the averaged intensities was then smoothed using a kernel density estimation method for visualization. This analysis was performed for both pre- and post-infection data across the respective groups. In addition, different plots were generated by subtracting the pre-infection values from the post-infection values.

### 2.3. Statistical Analysis

The unpaired *t*-test was used to compare continuous variables between groups, and the Wilcoxon signed-rank test was used to compare continuous variables before and after COVID-19 infection in the same groups. The Mann–Whitney U test was used to compare continuous variables between the two groups. Fisher’s exact test was utilized to compare comorbidities in each group. The Wilcoxon signed-rank test was performed using R version 4.1.1 using graphing software (Graph-R for Mac ver. 1.24.3, S-NEXT, Iwate, Japan).

## 3. Results

### 3.1. Study Populations

In total, 54 patients were diagnosed with COVID-19 infection via reverse transcription polymerase chain reaction. Then, they underwent blood sample collection before and during hospitalization. The following patients were excluded: one who received a blood transfusion during COVID-19, two who received granulocyte-colony stimulating factor, two who developed infections (febrile thrombophlebitis and febrile neutropenia), and one who exhibited exacerbation of macroglobulinemia. Thus, 48 patients were included in the analysis.

The patient distribution was as follows: 29 patients who did not receive immunosuppressive treatment for their underlying disease were in the control group, while 19 patients who received immunosuppressive treatment were in the IST group (Table 2 and Table 3). The underlying conditions in the IST group included malignancies, hematologic diseases, and autoimmune disorders. Approximately half of the IST group (12 out of 19) were treated with steroids (prednisolone and dexamethasone) (Table 1). The two groups did not significantly differ in clinical characteristics, such as age, sex, comorbidities, and COVID-19 severity at onset (Table 2 and Table 3).

The control group had higher CRP levels than the IST group. However, the two groups did not significantly differ in terms of the levels of several other inflammatory markers, such as ferritin and organ damage markers (e.g., lactate dehydrogenase, aspartate aminotransferase) (Table 3).

### 3.2. Baseline Characteristics Between the Control and IST Groups

The control and IST groups did not significantly differ in terms of total WBC counts and CPD before COVID-19 infection (Table 2). The control group had a high neutrophil count and a low lymphocyte count. The basophil and eosinophil counts of the two groups were hardly detectable, and the IST group had a significantly higher basophil count than the control group (*p* = 0.005) (Table 2).

### 3.3. Changes in CBC and CPD in the Control Group

The left side of Table 5 shows the serial changes in CBC and CPD in the control group. In particular, the WBC and neutrophil count of the control group did not significantly decrease immediately after symptom onset, followed by a recovery trend. The lymphocyte count of the control group decreased significantly immediately after disease onset (days 0–2) (*p* = 0.018). There was no remarkable change in the lymphocyte- and monocyte-related CPD. NE-FSC, a marker of cell size, significantly decreased immediately after onset (*p* = 0.009). NE-WX, which is the width of dispersion of neutrophil internal complexity, significantly increased after day 3 (*p* = 0.025). The monocyte counts did not significantly increase after the acute phase (days 3–5). The platelet count of the control group decreased significantly after day 3 (*p* = 0.037).

### 3.4. Changes in CBC and CPD in the IST Group

The right side of Table 5 shows the serial changes in CBC and CPD in the IST group. In particular, the WBC and neutrophil counts of the IST group did not significantly increase after onset. The IST group had significantly higher WBC and neutrophil counts immediately after COVID-19 onset than the control group (*p* = 0.043, *p* = 0.014, respectively). The lymphocyte count of the IST group, similar to that of the control group, also significantly decreased in the immediate phase (*p* = 0.004). However, significant recovery was delayed until days 6–8 (days 3–5 vs. days 6–8, 0.76 [0.65–1.21] vs. 1.49 [0.98–1.84], *p* = 0.034). LY-Y, which is the lymphocyte DNA/RNA content, did not significantly decrease after day 3. Thereafter, a recovery trend was observed (*p* = 0.020). LY-WY, which is the width of dispersion of the median values related to the lymphocyte DNA/RNA content, decreased after COVID-19 onset (*p* = 0.049). LY-WZ increased after day 3 (*p* = 0.034). The neutrophil count of the IST group did not significantly increase, which is in contrast to that of the control group after onset. NE-WY had a transient decrease after day 3 (*p* = 0.010). The monocyte count did not significantly increase after COVID-19 onset. MO-WY transiently increased after onset (*p* = 0.008) and decreased after day 3 (*p* = 0.017). The platelet counts decreased significantly after day 3 (*p* = 0.037).

### 3.5. WDF Scattergram of the Control and IST Groups 

In the WDF scattergram, the control and IST groups exhibited clusters of lymphocytes, monocytes, and neutrophils (Figure 2 and Figure 3). Immediately after COVID-19 infection, the lymphocyte cluster of the control and IST groups decreased, which is consistent with the cell count data presented above.

To analyze these changes in detail, subtraction images comparing pre-onset and immediate post-onset data were generated (Figure 2). The control and IST groups showed a blue color, indicating a decrease in the lymphocyte cluster, with the IST group presenting with a more pronounced reduction in juvenile lymphocytes. In the monocyte area, a small cluster of immature cells increased in the control group. Meanwhile, a more mature cluster expanded in the IST group.

In the neutrophil area, the control group exhibited a significant decrease in mature neutrophil clusters and an increase in immature neutrophil clusters. In contrast, the IST group presented with an increase in the mature neutrophil cluster and a slight decrease in the immature cluster.

In addition, discontinuous plasmacytoid lymphocyte clusters, a feature specific to COVID-19 infection, located in the upper region of the scattergram were examined [5]. These clusters were absent before onset in both groups but appeared immediately after onset. The subtraction images revealed slight clusters of plasmacytoid lymphocytes in the control and IST groups in Figure 3. Then, the scale was adjusted to make it easier to visualize areas with small numbers.

## 4. Discussion

In this study, CPD parameters were selected based on their ability to reflect key aspects of immune cell activation, immaturity, and heterogeneity—features that are central to immune dysfunction in COVID-19. Specifically, SSC (X) reflects cellular granularity and internal complexity, SFL (Y) reflects nucleic acid content, and FSC (Z) reflects cell size. The corresponding distribution widths (WX, WY, WZ) provide insight into the diversity within each leukocyte population. These parameters were interpreted in conjunction with WDF scattergrams, which enabled a visual assessment of cluster shifts in lymphocytes, neutrophils, and monocytes. By analyzing both CPD and WDF scattergram data before and after infection, we aimed to comprehensively characterize leukocyte dynamics, with a particular focus on identifying the suppression of immature cell recruitment and altered activation patterns in patients who are immunosuppressed.

The immunological backgrounds of the patients in the immunosuppressive treatment (IST) group are highly heterogeneous, and this diversity must be taken into account when interpreting the results. As shown in Table 1, the IST group in our study included patients with various immunocompromised conditions, such as autoimmune diseases, hematologic malignancies, inflammatory disorders, solid tumors, and solid organ transplants. According to Bertini et al., these conditions impair immune function through distinct mechanisms [10]. For example, hematologic malignancies and hematopoietic cell transplantation may lead to lymphopenia and neutropenia through bone marrow suppression and cytotoxic treatment, resulting in cellular and humoral immunodeficiencies. In contrast, patients with solid organ transplants or autoimmune rheumatologic diseases undergoing immunosuppressive therapy may have suppressed T and B cell functions along with the potential impairment of innate immunity. These mechanistic differences likely contributed to the variations in leukocyte dynamics observed in the IST group, including differences in CBC, CPD parameters, and WDF scattergram patterns. Thus, when comparing the immune responses between the IST and control groups, it is essential to consider this immunological heterogeneity.

The serial changes in CBC, CPD, and WDF scattergrams before and after COVID-19 onset were compared between the control and IST groups. Most hematological data were comparable between the two groups at baseline. However, the immune response to SARS-CoV-2 infection was distinct. The control group had an increase in the mobilization of juvenile cells. Meanwhile, the IST group exhibited a decrease in juvenile cells and compensation by mature cells. The baseline (i.e., pre-infection) CBC and CPD counts, except for an increase in basophil counts, were comparable in the IST group (*p* = 0.023). This pre-infection elevation may be attributable to leukocyte mobilization associated with chronic inflammation in autoimmune diseases and malignancy [9]. The control and IST groups had significantly decreased platelet counts after day 3 of infection. In a previous study, thrombocytopenia was associated with a threefold increased risk of severe COVID-19 and an elevated risk of mortality [11]. The mechanisms underlying thrombocytopenia in COVID-19 are still under investigation; however, the major causes may include cytokine-induced platelet damage, direct viral effects, and immune-mediated destruction [12]. Furthermore, Qiu et al. demonstrated that, in severe COVID-19 cases, platelet subpopulations exhibited the upregulation of pathways associated with endothelial injury and disseminated intravascular coagulation (DIC), along with the downregulation of MHC class II gene expression and lymphocyte activation pathways. These findings suggest that platelets may also influence lymphocyte activation and differentiation, expanding their role in immune dysregulation during severe COVID-19 [13].

The decrease in lymphocyte count, similar CRP, ferritin, and organ damage markers such as LDH in patients with COVID-19 is a biomarker of disease severity [14]. In the current study, the control and IST groups presented with a decrease in lymphocyte count. However, this decrease was sustained over a longer period in the IST group (Table 5). Pozdnyakova et al. reported a continuous decline in absolute lymphocyte counts from intensive care unit (ICU) admission to death, with a more pronounced decline in patients requiring mechanical ventilation, especially those with poor outcomes [15]. The decrease in lymphocyte count is attributed to a reduction in all CD3+ T lymphocyte subsets and B lymphocytes, particularly in severe cases [16]. The delayed lymphocyte recovery in the IST group was related to the immunosuppressive state. Hence, it may serve as a prognostic factor. Patients with COVID-19 commonly present with diverse abnormal lymphocytes, often referred to as reactive, activated, or pleomorphic [17]. Previous studies by Harte et al. and Martens et al. reported increases in LY-FSC (cell size), LY-SSC (intracellular complexity), and RE-LYMPHO (reactive lymphocytes) in patients with COVID-19, which are considered indicators of lymphocyte activation in response to viral infection [18,19]. In our study, group- and time-specific changes were observed. In the control group, although no statistically significant CPD changes were noted, the subtraction images of the WDF scattergram during days 0–2 showed an increase in juvenile lymphocyte clusters, suggesting the mobilization of reactive lymphocytes. In contrast, in the IST group, LY-WY significantly decreased on days 0–2 (*p* = 0.049), and the scattergram revealed a broad reduction in lymphocytes and cluster shrinkage. These findings suggest suppressed early lymphocyte mobilization and reduced structural diversity in the IST group. Furthermore, LY-Y significantly increased on days 3–5 (*p* = 0.020), and LY-WZ also fluctuated, suggesting increased nucleic acid content and cell size heterogeneity, which may reflect a delayed and heterogeneous adaptive immune response under immunosuppressive conditions. Taken together, these results suggest that early-phase lymphocyte mobilization and activation were suppressed in patients who were immunosuppressed, possibly due to underlying diseases and immunosuppressive therapies that attenuate T cell responses. Lymphopenia in COVID-19 is attributed to impaired T cell circulation, which leads to their depletion from the peripheral blood. This is driven by the migration of T lymphocytes to infected tissues, such as those in the lungs, in response to chemokine signals. At these sites, T cells can become overactivated, fail to control the virus, and, ultimately, undergo cell death via apoptosis or pyroptosis [20]. In the IST group, steroids, immunosuppressive drugs, and anticancer drugs might have suppressed the mobilization and migratory capacity of juvenile T cells, contributing to the reduction in T cells due to SARS-CoV-2 infection [2]. In this study, the two groups presented with discontinuous plasmacytoid lymphocyte clusters, a feature specific to COVID-19 infection (Figure 3) [5]. However, their cellular appearance is not correlated with disease severity [21], and their pathogenesis is unknown.

Neutrophilia is a sign of adverse prognosis in COVID-19 [22]. The transient decrease in neutrophil counts in the control group along with the increasing trend and higher neutrophil count in the IST group (Table 5) might suggest a poorer prognosis in the IST group. Previous studies have reported increased NE-SFL and decreased NE-WY in patients with COVID-19, reflecting neutrophil activation, immaturity, and heterogeneity [18,19]. Additionally, Rutkowska et al. reported that a decrease in NE-FSC may indicate neutrophil degranulation and structural exhaustion in the context of COVID-19 [23]. In our study, group- and time-dependent changes were also observed. In the control group, NE-FSC significantly decreased on days 0–2 (*p* = 0.009), suggesting morphological shrinkage and exhaustion due to early neutrophil activation. NE-WX significantly increased on days 3–5 (*p* = 0.025), suggesting the recruitment of immature neutrophils during early infection. These changes were corroborated by scattergram findings showing a reduction in mature neutrophil clusters and an increase in immature clusters, consistent with a left shift involving band cells, myelocytes, metamyelocytes, and promyelocytes [19]. In contrast, in the IST group, no significant decrease in NE-FSC was observed during the early phase, but NE-WY significantly decreased on days 3–5 (*p* = 0.010). Scattergram analysis revealed an expansion of mature neutrophil clusters and a reduction in immature clusters, suggesting that early neutrophil activation and the mobilization of immature forms were both suppressed. Consequently, the peripheral neutrophil population in patients who were immunosuppressed appeared to be skewed toward a more homogeneous, mature-dominant profile. Based on these results, mature neutrophils were consumed by inflammation. Meanwhile, juvenile neutrophils were mobilized in response to cytokine stimulation. Insufficient early type I interferon responses can allow unchecked viral replication during the early onset of COVID-19, leading to an exaggerated release of pro-inflammatory cytokines, such as interleukin-6, interleukin-1β, and tumor necrosis factor alpha, resulting in the recruitment of neutrophils [24]. Anti-cytokine therapy and anticancer drugs could reduce cytokine-induced neutrophil recruitment to the bone marrow in the IST group. Furthermore, 12 of the 19 patients in the IST group had received steroids prior to the disease (Table 1). Steroid administration increases the proportion of mature neutrophils in the circulation by reducing their adhesion to the vessel walls via the downregulation of integrins and selectins and the decreased migration of neutrophils from the blood to the tissue [25,26]. Notably, the scattergram in the IST group showed an increase in the mature neutrophil cluster and a decrease in the immature cluster (Figure 2). Immature neutrophils could mediate important innate immune functions such as bacterial phagocytosis and killing via the production of reactive oxygen species; however, they are less efficient than mature neutrophils [27]. The decrease in immature neutrophils may cause a blunted immune response despite the increase in neutrophil counts in the IST group.

The reported changes in monocyte counts after SARS-CoV-2 infection vary; that is, they can increase and decrease [17]. Previous studies have reported increased MO-SFL (monocyte fluorescence intensity) and MO-SSC (side scatter intensity) in patients with COVID-19, which are considered indicators of increased membrane permeability and enhanced intracellular structural activity, such as vacuolization and granulation [19]. In our study, no statistically significant changes in monocyte-related CPD parameters were observed in the control group. However, the subtraction images of the WDF scattergram during days 0–2 revealed the emergence of immature monocyte clusters, suggesting monocyte activation during the early phase of infection (Figure 2). In contrast, the IST group demonstrated distinct changes. MO-WY (width of MO-SFL) significantly increased during days 0–2 (*p* = 0.008), suggesting the presence of activated monocytes with diverse DNA/RNA content [15]. However, the subtraction images of the scattergram revealed a marked expansion of mature monocyte clusters, while immature clusters were only minimally present. This indicates that the monocyte population was structurally skewed toward maturity during the early phase of infection. Subsequently, MO-WY significantly decreased during days 3–5 (*p* = 0.017), suggesting that monocytes initially activated in the early phase were later suppressed or functionally exhausted. The underlying mechanism could be the suppression of cytokines by steroids and immunosuppressive drugs as well as monocyte and bone marrow suppression caused by anticancer drugs.

In particular, CPD and WDF scattergrams provide a non-invasive and continuous means of monitoring the intracellular characteristics of leukocytes, such as nucleic acid content, structural complexity, and heterogeneity. These parameters are automatically obtained from routine CBC testing without requiring additional procedures or costs, making them highly practical in clinical settings. In this study, we demonstrated that specific CPD and WDF scattergram indices, particularly those related to neutrophils, lymphocytes, and monocytes, showed distinct alterations from the early phase of COVID-19 onset, especially in patients who were immunosuppressed. These changes, such as the impaired mobilization of immature cells, the dominance of mature subpopulations, and reduced diversity within leukocyte clusters, may reflect qualitative immune dysfunction and delayed immune recovery. As such, CPD and WDF scattergram analyses may offer practical utility in the following areas: (1) immune monitoring, by enabling the real-time and longitudinal tracking of leukocyte functional states, and (2) therapeutic decision-making, by assisting clinicians in determining the optimal timing for initiating antiviral or antibacterial therapy, particularly in patients who are immunocompromised and at higher risk of secondary infections. These findings support the potential clinical value of CPD-based immune profiling in managing COVID-19 under immunosuppressed conditions.

To the best of our knowledge, this is the first study to longitudinally assess the changes in leukocyte CPD and WDF scattergrams before and after COVID-19 onset in the same individuals. Furthermore, by comparing patients who were immunocompromised and receiving immunosuppressive therapy (IST group) with non-immunosuppressed controls, we were able to identify distinct patterns in immune cell dynamics. This study provides novel insights into immune responses to COVID-19 under immunosuppressive conditions.

This study has several limitations. First, it was a retrospective observational study conducted at a single center. In addition, the relatively small sample size (n = 48) may have reduced the statistical power. Second, the immunosuppressed treatment group (IST group) included patients with a wide range of underlying diseases (e.g., malignancy, autoimmune diseases, and post-transplantation) and treatment regimens (e.g., corticosteroids, immunomodulators, and anticancer agents), which made it difficult to fully control for confounding factors that might influence leukocyte dynamics. Third, vaccination history could not be obtained from the medical records and was therefore not considered in this study. However, in Japan, the vaccination rate for up to three doses is high, ranging from 65% to 80%, according to a survey by the Ministry of Health, Labour and Welfare [24]. Given these limitations, further prospective, multi-center studies with larger sample sizes are warranted to validate our findings.

In addition, it should be noted that the CPD (cell population data) parameters used in this study do not have officially established reference ranges for healthy individuals. CPD represents raw measurements that are highly dependent on the optical and analytical design of the hematology analyzer employed. As a result, standardization across different instruments is lacking, making it difficult to propose unified reference values applicable across platforms [28]. Each parameter requires instrument-specific clinical decision limits, and the absence of harmonization between devices and laboratories further limits their generalizability. Because the measurement principles and signal processing algorithms vary across different analyzers, the harmonization of CPD values between different platforms is inherently difficult. Analytical quality specifications, such as stability, quality control targets, and the harmonization of CPDs, remain unresolved metrological issues and are currently under active evaluation [28,29,30].

## 5. Conclusions

IST could impair the recruitment of immature leucocytes and relatively increase the proportion of mature leucocytes. These mechanisms might reduce cytokine induction and blunt the immune response, resulting in potentially worse outcomes. Thus, further research should be performed to optimize preventive strategies, such as novel vaccinations and the use of monoclonal antibodies, to enhance passive immunity and other treatments, which include safer antiviral therapy, for individuals with compromised immune systems [2].

## Figures and Tables

**Table 1:** Background diseases and therapeutic agents in the immunosuppressed patient (IST) group.

No.	Background Diseases	Immunosuppressive Therapy
1	Myasthenia gravis	Prednisolone
2	Neuro-sweet disease	Prednisolone
3	Interstitial lung disease	Prednisolone
4	Encephalitis	Prednisolone
5	Eosinophilic granulomatosis with polyangiitis	Prednisolone
6	Diffuse large B cell lymphoma	R-CHOP
7	Multiple myeloma	Bortezomib, lenalidomide
8	Multiple myeloma	Bortezomib, dexamethasone
9	POEMS syndrome	Dexamethasone, lenalidomide
10	Essential thrombocythemia	Ruxolitinib
11	Rheumatoid arthritis	Infliximab, methotrexate
12	Microscopic polyangiitis, rapid progressive glomerulonephritis	Azathioprine, prednisolone
13	Crohn’s disease	Infliximab
14	Crohn’s disease	Azathioprine, infliximab, mesalazine
15	Ulcerative colitis	Betamethasone (enema), mesalazine
16	Pancreatic cancer	S-1
17	Pancreatic cancer	S-1
18	Lung cancer	Durvalumab, prednisolone
19	After liver transplantation	Cyclosporine, everolimus, methylprednisolone

This table shows the background disease of the IST group and the immunosuppressive therapy used upon COVID-19 onset.

**Table 2:** Baseline characteristics of the study population.

	Control Group		Immunosuppressive Treatment (IST) Group		*p*-Value
	n = 29		n = 19		
Age (mean, year)	65.7 ± 13.17		60.3 ± 17.11		0.226
Male sex—no. (%)	20 (69.0%)		10 (52.6%)		0.362
BMI (mean, kg/m^2^)	25.4 ± 4.90		23.9 ± 3.54		0.27
Comorbidities, at least one, number (%)					
Hypertension	15 (51.7%)		8 (42.1%)		0.566
Diabetes mellitus	10 (34.5%)		5 (26.3%)		0.751
Obesity (BMI > 25 kg/m^2^)	13 (46.4%)		7 (36.8%)		0.561
COPD	4 (13.8%)		1 (5.3%)		0.635
Asthma	0 (0%)		2 (10.5%)		0.152
Chronic heart disease	3 (10.3%)		1 (5.3%)		1
Chronic kidney disease	9 (31.0%)		3 (15.8%)		0.316
Baseline complete blood count (CBC) and cell population data (CPD) before COVID-19 onset—median [25th–75th percentile]					
WBC count (10^3^/μL)	5.68	[4.43–5.96]	5.11	[4.73–7.64]	0.706
HGB level (g/dL)	13.0	[12.4–13.9]	12.7	[12.0–13.3]	0.333
HCT count (%)	38.8	[37.8–40.1]	39.5	[34.9–40.3]	0.706
PLT count (10^4^/μL)	174	[135–224]	208	[126–274]	0.568
NEUT# count (10^3^/μL)	3.42	[2.25–4.46]	2.67	[2.55–3.54]	0.716
LYMPH# count (10^3^/μL)	1.15	[1.07–1.85]	1.79	[1.36–1.98]	0.303
MONO# count 10^3^/μL)	0.30	[0.19–0.35]	0.31	[0.30–0.37]	0.220
EO# count (10^3^/μL)	0.07	[0.02–0.12]	0.11	[0.10–0.17]	0.131
BASO# count (10^3^/μL)	0.02	[0.01–0.03]	0.05	[0.03–0.05]	0.005 *
NE-SSC (ch)	150	[149–151]	148	[144–153]	0.637
NE-SFL (ch)	47.9	[46.2–48.3]	47.2	[46.0–51.1]	0.608
NE-FSC (ch)	88.5	[86.7–90.8]	86.6	[84.5–89.3]	0.160
NE-WX	310	[300–325]	316	[310–326]	0.357
NE-WY	623	[621–648]	646	[629–679]	0.221
NE-WZ	685	[631–834]	669	[603–794]	0.457
LY-X (ch)	79.4	[78.1–81.5]	80.8	[79.5–82.1]	0.303
LY-Y (ch)	66.5	[66.4–70.4]	66.4	[64.3–67.3]	0.157
LY-Z (ch)	58.8	[57.0–61.1]	59.1	[57.8–60.0]	0.935
LY-WX	516	[477–521]	476	[391–529]	0.196
LY-WY	813	[796–814]	821	[799–878]	0.316
LY-WZ	608	[538–634]	533	[484–646]	0.237
MO-X (ch)	121	[118–121]	121	[118–121]	0.829
MO-Y (ch)	110	[108–112]	115	[108–116]	0.060
MO-Z (ch)	68.2	[66.3–74.4]	69.3	[65.2–71.3]	0.394
MO-WX	286	[267–290]	284	[242–292]	0.364
MO-WY	665	[526–736]	684	[622–703]	0.871
MO-WZ	632	[609–633]	606	[571–695]	0.526

Data are presented as the mean, number of patients (%), and median (25th–75th percentile). Unpaired *t*-tests were used to compare continuous variables between the control and immunosuppressive treatment (IST) group. Fisher’s exact test was utilized to compare comorbidities between groups. The Wilcoxon signed-rank test was used to compare continuous variables between the control and immunosuppressive treatment (IST) group. * *p* < 0.05 indicates statistically significant differences.

**Table 3:** Comparison of COVID-19 severity and associated factors at the time of hospital admission.

	Control Group	Immunosuppressive Treatment (IST) Group	*p*-Value
	n = 29	n = 19	
Severity—no. (%)			
Mild	21 (72.4%)	13 (68.4%)	1
Severe	8 (27.6%)	6 (31.6%)	
Variable severity factors (including inflammatory variables)—median [25th–75th percentile]			
CRP level (mg/dL)	2.11 [1.24–4.19]	0.77 [0.30–3.98]	0.077
Procalcitonin level (ng/mL)	0.10 [0.10–0.20]	0.10 [0.10–0.20]	0.875
Ferritin level (ng/mL)	274 [126–499]	312 [251–428]	0.386
LD level (units/L)	272 [224–340]	250 [192–339]	0.424
AST level (units/L)	35.0 [25.8–45.5]	31.5 [22.0–45.8]	0.485
ALT level (units/L)	25.0 [16.0–43.0]	24.0 [17.8–29.3]	0.677
eGFR (mL/min/1.73 m^2^)	69.1 [51.3–80.0]	66.9 [54.0–75.5]	0.768

Data are presented as the mean, severity (%), and median (25th–75th percentile). Fisher’s exact test was used to compare COVID-19 severity between each group, and the Mann–Whitney U test was used to compare continuous variables between the two groups. A *p*-value < 0.05 indicates statistically significant differences. Mild cases are defined as the absence of respiratory symptoms or pneumonia findings, with oxygen saturation > 93% in room air and no requirement for oxygen therapy. Severe cases are defined as respiratory failure, an oxygen saturation level < 93% in room air, and a clinical requirement for oxygen administration [7].

**Table 4:** Morphological and functional characteristics of the CPD in Sysmex XN-9000^®^.

Parameters	Parameter Description	Method Used to Analyze Clusters
NE-SSC (ch)	Complexity of the intracellular structure of the neutrophils (intracellular structure and granularity)	The laterally scattered light intensity of the neutrophil area on the WDF scattergram
NE-SFL (ch)	DNA/RNA content indicating cell immaturity or activation	The fluorescent light intensity of the neutrophil area on the WDF scattergram
NE-FSC (ch)	Size or volume of neutrophils	The forward-scattered light intensity of the neutrophil area on the WDF scattergram
NE-WX	Dispersion of the NE-SSC signal of the neutrophils	The laterally scattered light distribution width index of the neutrophil area on the WDF scattergram
NE-WY	Dispersion of the NE-SFL signal of the neutrophils	The fluorescent light distribution width index of the neutrophil area on the WDF scattergram
NE-WZ	Dispersion of the NE-FSC signal of the neutrophils	The forward-scattered light distribution width index of the neutrophil area on the WDF scattergram
LY-X (ch)	Complexity of the intracellular structure of the lymphocytes (e.g., nuclear irregularities and vacuolation)	The laterally scattered light intensity of the lymphocyte area on the WDF scattergram
LY-Y (ch)	DNA/RNA content indicating cell immaturity or activation	The fluorescent light intensity of the lymphocyte area on the WDF scattergram
LY-Z (ch)	Size or volume of the lymphocytes	The forward-scattered light intensity of the lymphocyte area on the WDF scattergram
LY-WX	Dispersion of the LY-X signal of the lymphocytes	The laterally scattered light distribution width index of the lymphocyte area on the WDF scattergram
LY-WY	Dispersion of the LY-Y signal of the lymphocytes	The fluorescent light distribution width index of the lymphocyte area on the WDF scattergram
LY-WZ	Dispersion of the LY-Z signal of the lymphocytes	The forward-scattered light distribution width index of the lymphocyte area on the WDF scattergram
MO-X (ch)	Complexity of the intracellular structure of the monocytes (e.g., nuclear irregularities and vacuolation)	The laterally scattered light intensity of the monocyte area on the WDF scattergram
MO-Y (ch)	DNA/RNA content indicating cell immaturity or activation	The fluorescent light intensity of the monocyte area on the WDF scattergram
MO-Z (ch)	Size or volume of the monocytes	The forward-scattered light intensity of the monocyte area on the WDF scattergram
MO-WX	Dispersion of the MO-X signal of the monocytes	The laterally scattered light distribution width index of the monocyte area on the WDF scattergram
MO-WY	Dispersion of the MO-Y signal of the monocytes	The fluorescent light distribution width index of the monocyte area on the WDF scattergram
MO-WZ	Dispersion of the MO-Z signal of the monocytes	The forward-scattered light distribution width index of the monocyte area on the WDF scattergram

**Table 5:** Serial changes in complete blood count (CBC) data and cell population data (CPD) before and after COVID-19 onset.

	Control Group								Immunosuppressive Treatment (IST) Group							
	Median [25th–75th Percentile]						*p*-Value		Median [25th–75th Percentile]						*p* Value	
	Before		Days 0–2		Days 3–5		Before	Days 0–2	Before		Days 0–2		Days 3–5		Before	Days 0–2
							vs.	vs.							vs.	vs.
							Days 0–2	Days 3–5							Days 0–2	Days 3–5
WBC count (10^3^/μL)	5.68	[4.43–5.96]	3.91	[3.63–5.93]	4.43	[3.94–6.50]	0.317	0.174	5.11	[4.73–7.64]	5.3	[4.87–7.73]	5.97	[3.93–6.93]	0.595	0.583
NEUT count (10^3^/μL)	3.42	[2.25–4.46]	2.41	[2.05–2.67]	2.88	[2.10–5.02]	0.096	0.335	2.67	[2.55–3.54]	3.89	[3.37–5.66]	3.49	[2.45–5.14]	0.073	0.429
LYMPH# count (10^3^/μL)	1.15	[1.07–1.85]	0.98	[0.86–1.13]	1.28	[1.01–2.00]	0.018 *	0.082	1.79	[1.36–1.98]	0.73	[0.54–1.23]	0.76	[0.65–1.21]	0.004 *	0.78
MONO# count (10^3^/μL)	0.3	[0.19–0.35]	0.33	[0.35–0.40]	0.45	[0.32–0.54]	0.204	0.024 *	0.31	[0.30–0.37]	0.42	[0.35–0.65]	0.47	[0.25–0.56]	0.105	0.653
HGB level (g/dL)	13	[12.4–13.9]	12.7	[11.8–0.40]	13.7	[12.4–15.0]	0.494	0.15	12.7	[12.0–13.3]	13.2	[13.0–13.5]	13.1	[10.1–14.8]	0.322	0.815
HCT count (%)	38.8	[37.8–40.1]	36.9	[35.3–42.9]	39.9	[37.4–44.7]	0.45	0.075	39.5	[34.9–40.3]	40.2	[37.7–40.6]	40.4	[30.9–43.6]	0.467	0.96
PLT count (10^4^/μL)	174	[135–224]	189	[119–224]	137	[112–181]	0.963	0.037 *	208	[126–274]	205	[133–237]	105	[98.0–189]	0.785	0.037 *
NE-SSC (ch)	150	[149–151]	151	[148–152]	152	[149–155]	0.369	0.584	148	[144–153]	149	[149–159]	155	[149–158]	0.112	0.653
NE-SFL (ch)	47.9	[46.2–48.3]	47	[46.0–48.4]	49	[46.9–51.1]	0.919	0.353	47.2	[46.0–51.1]	47.1	[44.0–51.6]	50.4	[48.1–52.2]	0.784	0.125
NE-FSC (ch)	88.5	[86.7–90.8]	85.7	[84.5–88.0]	87.6	[85.4–89.8]	0.009 *	0.167	86.6	[84.5–89.3]	88.3	[86.4–90.5]	88.2	[84.9–90.4]	0.356	0.78
NE-WX	310	[300–325]	307	[304–318]	325	[317–334]	0.433	0.025 *	316	[310–326]	317	[301–339]	310	[298–330]	0.885	0.618
NE-WY	623	[621–648]	645	[631–658]	650	[624–672]	0.345	0.713	646	[629–679]	659	[637–680]	623	[607–638]	0.784	0.010 *
NE-WZ	685	[631–834]	669	[639–715]	647	[616–742]	0.345	0.713	669	[603–794]	643	[605–710]	658	[600–720]	0.664	0.799
LY-X (ch)	79.4	[78.1–81.5]	80	[78.2–81.7]	77.3	[74.5–79.9]	0.812	0.059	80.8	[79.5–82.1]	77.5	[75.9–81.2]	79.3	[76.2–83.7]	0.137	0.246
LY-Y (ch)	66.5	[66.4–70.4]	68.7	[66.0–69.7]	67.3	[65.0–69.7]	0.533	0.382	66.4	[64.3–67.3]	64.1	[62.4–67.1]	68.4	[65.2–70.6]	0.447	0.020 *
LY-Z (ch)	58.8	[57.0–61.1]	57.9	[57.0–59.4]	57.8	[56.8–58.9]	0.548	0.726	59.1	[57.8–60.0]	57.6	[56.9–58.4]	59.6	[57.6–60.6]	0.21	0.166
LY-WX	516	[477–521]	558	[444–586]	534	[477–589]	0.24	0.66	476	[391–529]	477	[456–549]	552	[480–609]	0.392	0.251
LY-WY	813	[796–814]	867	[809–926]	809	[732–589]	0.24	0.134	821	[799–878]	776	[678–820]	797	[726–895]	0.049 *	0.312
LY-WZ	608	[538–634]	586	[498–630]	560	[538–613]	0.489	0.979	533	[484–646]	525	[498–552	583	[553–605]	0.885	0.034 *
MO-X (ch)	121	[118–121]	122	[120–123]	121	[119–122]	0.139	0.229	121	[118–121]	120	[118–123]	121	[120–123]	0.834	0.3
MO-Y (ch)	110	[108–112]	114	[104–122]	112	[109–117]	0.3	0.957	115	[108–116]	106	[99.0–116]	108	[105–113]	0.322	0.544
MO-Z (ch)	68.2	[66.3–74.4]	66.9	[64.0–69.0]	66.2	[65.2–67.4]	0.111	0.822	69.3	[65.2–71.3]	66.5	[63.7–69.9]	67.6	[64.7–69.7]	0.339	0.837
MO-WX	286	[267–290]	267	[247–292]	261	[247–276]	0.334	0.686	284	[242–292]	269	[250–287]	265	[243–290]	0.988	0.636
MO-WY	665	[526–736]	696	[580–740]	725	[683–745]	0.503	0.321	684	[622–703]	775	[720–813]	664	[603–708]	0.008 *	0.017 *
MO-WZ	632	[609–633]	620	[561–650]	668	[581–709]	0.563	0.226	606	[571–695]	605	[595–642]	617	[586–694]	0.688	0.904

Data are presented as the median (25th–75th percentile) in patients in each period. The Wilcoxon signed-rank test was performed in each period, with * *p* < 0.05. The “Before” data partially overlap with the values presented in Table 2 under “Baseline complete blood count (CBC) and cell population data (CPD) before COVID-19 onset—median [25th–75th percentile]”; however, they are presented again due to differences in purpose and to ensure readability.

## Data Availability

The (primary) data that support the findings of this study are not publicly available due to their containing information that could compromise the privacy of research participants but are available from the corresponding authors, Kyosuke Takeshita and Masumi Ogawa, upon reasonable request.

## Abbreviations

The following abbreviations are used in this manuscript:

BMI	body mass index
COPD	chronic obstructive pulmonary disease
WBC	white blood cell count
HGB	hemoglobin concentration
HCT	hematocrit
PLT	absolute number of thrombocytes
NEUT#	neutrophil count
LYMPH#	lymphocyte count
MONO#	monocyte count
EO#	eosinophilic count
BASO#	basophilic count
NE-SSC	neutrophil complexity
NE-SFL	neutrophil fluorescence
NE-FSC	neutrophil size
NE-WX	width of dispersion of neutrophil complexity
NE-WY	width of dispersion of neutrophil fluorescence
NE-WZ	width of dispersion of neutrophil size
LY-X	lymphocyte complexity
LY-Y	lymphocyte fluorescence
LY-Z	lymphocyte size
LY-WX	width of dispersion of lymphocyte complexity
LY-WY	width of dispersion of lymphocyte fluorescence
LY-WZ	width of dispersion of lymphocyte size
MO-X	monocyte complexity
MO-Y	monocyte fluorescence
MO-Z	monocyte size
MO-WX	width of dispersion of monocyte complexity
MO-WY	width of dispersion of monocyte fluorescence
MO-WZ	width of dispersion of monocyte size
R-CHOP	rituximab, doxorubicin, vincristine, cyclophosphamide, prednisolone
S-1	tegaful/gimeracil/oteracil potassium
CRP	C-reactive protein
LD	lactate dehydrogenase
AST	aspartate transaminase
ALT	alanine transaminase
eGFR	estimated glomerular filtration rate
